# Integrated machine learning algorithms reveal a bone metastasis-related signature of circulating tumor cells in prostate cancer

**DOI:** 10.1038/s41597-024-03551-2

**Published:** 2024-06-27

**Authors:** Congzhe Ren, Xiangyu Chen, Xuexue Hao, Changgui Wu, Lijun Xie, Xiaoqiang Liu

**Affiliations:** Department of Urology, Tianjin Medical University General Hospital, Tianjin, China

**Keywords:** Tumour biomarkers, Cancer genomics

## Abstract

Bone metastasis is an essential factor affecting the prognosis of prostate cancer (PCa), and circulating tumor cells (CTCs) are closely related to distant tumor metastasis. Here, the protein-protein interaction (PPI) networks and Cytoscape application were used to identify diagnostic markers for metastatic events in PCa. We screened ten hub genes, eight of which had area under the ROC curve (AUC) values > 0.85. Subsequently, we aim to develop a bone metastasis-related model relying on differentially expressed genes in CTCs for accurate risk stratification. We developed an integrative program based on machine learning algorithm combinations to construct reliable bone metastasis-related genes prognostic index (BMGPI). On the basis of BMGPI, we carefully evaluated the prognostic outcomes, functional status, tumor immune microenvironment, somatic mutation, copy number variation (CNV), response to immunotherapy and drug sensitivity in different subgroups. BMGPI was an independent risk factor for disease-free survival in PCa. The high risk group demonstrated poor survival as well as higher immune scores, higher tumor mutation burden (TMB), more frequent co-occurrence mutation, and worse efficacy of immunotherapy. This study highlights a new prognostic signature, the BMGPI. BMGPI is an independent predictor of prognosis in PCa patients and is closely associated with the immune microenvironment and the efficacy of immunotherapy.

## Introduction

Prostate cancer (PCa) is the second most prevalent tumor and the leading cause of mortality in men worldwide^1^. Bone is one of the most common sites of PCa metastasis. There are up to 90% of patients with advanced PCa who have metastases to their bones and nearly all patients who die from PCa exhibit signs of bone metastases^2^. Until now, endocrine therapy, radiation therapy, immunotherapy, and targeted therapy have become the mainstream therapies for metastatic prostate cancer. However, tumor heterogeneity and treatment resistance remain important barriers limiting durable efficacy^3,4^. Over recent years, a large number of prognostic markers for PCa have been identified by mining public databases for clinical and sequencing information. Despite their great potential, the accuracy needs to be further validated in real-world cohorts^5^. Therefore, it is necessary to take more scientific and standardized approaches to continue mining valuable prognostic signatures for PCa.

Circulating tumor cells (CTCs) are tumor cells that enter the human peripheral blood, and their metastatic colonization is a sentinel process of tumor metastasis^6^. According to extant research, epithelial mesenchymal transition (EMT) is the main driver for the generation of CTCs^7^. Interestingly, although the vast majority of CTCs die of programmed cell death (PCD) before leaving the vascular system, a small number of CTCs survive by counteracting apoptosis with the assistance of upregulated WNT signaling^8,9^. In recent years, with the gradual maturation of liquid biopsy technology, the scarce CTCs in the blood can now be isolated by a variety of methods, such as immunomagnetic enrichment, microfluidic sorting, and high content scanning^10^. These methods provide the condition for further sequencing of CTCs, thus pushing us to find more precise and sensitive biomarkers to improve prognosis.

In this work, using single cell sequencing data of CTCs, we attempted to construct a superior bone metastasis-related genes prognostic index (BMGPI) by a combination of batch machine learning algorithms to assess the prognosis, immunological and mutational landscapes, and response to immunotherapy in PCa patients. This study may contribute to the optimization of individualized treatment regimens and further improve survival outcomes for PCa patients.

## Materials and methods

### Data of patients and samples

Data of CTC and bone metastasis samples of PCa were obtained from the datasets GSE67980^11^ and GSE32269^12^, respectively, from the GEO database (http://www.ncbi.nlm.nih.gov/geo/). Information on the TCGA-PRAD cohort was acquired from the TCGA database (https://portal.gdc.cancer.gov/)^13^. Clinical and sequencing data for the MSKCC cohort were available from the cBioPortal database (https://www.cbioportal.org/study/summary?id=msk_impact_2017)^14^. Disease-free survival (DFS) was used as the survival endpoint of this study. Screening for differentially expressed genes (DEGs) was realized with the “limma” R package (|logFC| > 1, *p*. adj < 0.05).

### Functional enrichment and immune cell infiltration

The Gene Ontology (GO) database categorizes gene function into three sections: biological process (BP), cellular component (CC), and molecular function (MF). GO analysis can get what our target genes are related to at the BP, CC, and MF levels. Similarly, Kyoto Encyclopedia of Genes and Genomes (KEGG) analysis is the annotation of pathways in which target genes are involved. Both gene set enrichment analysis (GSEA) and gene set variation analysis (GSVA) are genome enrichment methods. The former is used to identify whether a pre-defined gene set is significantly different between two biological states. The latter converts gene expression data from the level of individual genes to the enrichment level of gene sets by calculating the enrichment level of gene sets in each sample. The principle of immune infiltration analysis is based on gene expression data, where the relative proportions of immune cell sub-populations in each sample are inferred from the gene expression labels of various immune cell types. The ESTIMATE method infers the proportion of stromal and immune cells in the tumor sample from Stromal signature and Immune signature.

GO analysis was achieved by uploading gene names to the Database for Annotation, Visualization, and Integrated Discovery (DAVID)^15^. We utilized GSEA to screen the functional pathways with the help of GSEA software^16^. The single-sample gene set enrichment analysis (ssGSEA) and GSVA were carried out using the R packages “GSVA” and “GSEABase” to score immune cell abundance and signaling pathways for each sample. The GSVA analysis process was referenced in gene set “c2.cp.kegg.v7.5.1.symbols” from the MSigDB (https://www.gsea-msigdb.org/gsea/msigdb/)^17^. We downloaded the reference file “infiltration_estimation_for_tcga” from the TIMER 2.0 (http://timer.cistrome.org/) to provide a data base for the immune cell infiltration analysis^18^. The bubble plot and heatmap of immune cells were made with the assistance of “ggplot2” and “pheatmap” R packages. Additionally, we calculate tumor microenvironment (TME) scores for risk groups using the R package “estimate”.

### Protein–protein interaction (PPI) network of BMRGs

The PPI network of 120 bone metastasis-related genes (BMRGs) was first constructed through the STRING database (http://string-db.org/)^19^. Subsequently, cytoHubba and MCODE plugins in Cytoscape software (https://cytoscape.org) were utilized to further screen the hub gene modules^20^. The diagnostic efficacy of hub genes was identified in GSE32269^12^ and GSE6919^21^ datasets.

### Construction of the bone metastasis-related genes prognostic index (BMGPI) based on combined machine learning

Firstly, univariate Cox analysis was applied to screen potential prognostic genes from 120 BMRGs (*p* < 0.001). Then, the TCGA-PRAD cohort was set as the training set, and the GSE46602^22^ and MSKCC cohorts were set as the external validation set. To develop more superior model, 94 combinations were constructed based on ten machine learning algorithms, including Lasso, random survival forest (RSF), stepwise Cox, Ridge, CoxBoost, elastic net (Enet), generalized boosted regression modeling (GBM), partial least squares regression for Cox (plsRcox), survival support vector machine (survival-SVM), and supervised principal components (SuperPC). 94 combinations of 10 algorithms were arranged to fit a variety of models in the training cohort. All constructed models were assessed in external validation cohorts. We calculated the concordance index (C-index) across all models for each cohort and ranked the models in terms of efficacy according to the average C-index of the three cohorts from highest to lowest.

The training and validation groups were categorized into low- and high-risk groups based on the median risk score (BMGPI). The R package “survminer” was applied for KM survival analysis. We then used the “timeROC” package to plot time-dependent receiver operator characteristic (ROC) curves to assess the predictive efficacy of BMGPI for survival.

### A predictive nomogram and survival analysis

Combining risk levels, Gleason scores, and T stage, we developed a nomogram relying on the “rms” package. Calibration plot, ROC curves, and C-index were employed to assess the accuracy of the nomogram.

### Somatic mutation and prediction for immunotherapy

The processing and visualization of mutation data and the calculation of tumor mutational burden (TMB) relied on the “maftools” R package. Subsequently, we selected differential genes between risk groups for analysis of copy number variation (CNV).

The Tumor Immune Dysfunction and Exclusion (TIDE) online site (http://tide.dfci.harvard.edu/) allows for the assessment of specific transcriptomic biomarkers to predict a patient’s response to treatment based on pre-treatment tumor expression profiles^23^. A higher TIDE score indicates a higher likelihood of tumor immune escape. TIDE scores for samples were generated from this online site. The chi-squared test was applied to compare the differences in the proportions of response to immunotherapy. The IMvigor210 cohort was used to test the predictive power of BMGPI for immunotherapy response. In addition, we introduced Immunophenoscore (IPS) scores for each patient sample. Higher IPS scores imply better immunotherapy efficacy. The Cancer Immunome Atlas (TCIA) database (https://tcia.at/home) provides IPS scores for patients in the TCGA-PRAD cohort^24^.

### Drug sensitivity

Drug sensitivity analysis in risk groups was conducted by the half-maximal inhibitory concentrations (IC50) on Genomics of Drug Sensitivity in Cancer (GDSC) (https://www.cancerrxgene.org/)^25^.

## Results

### Identification of differential genes related to bone metastasis in PCa

**Fig. 1:**
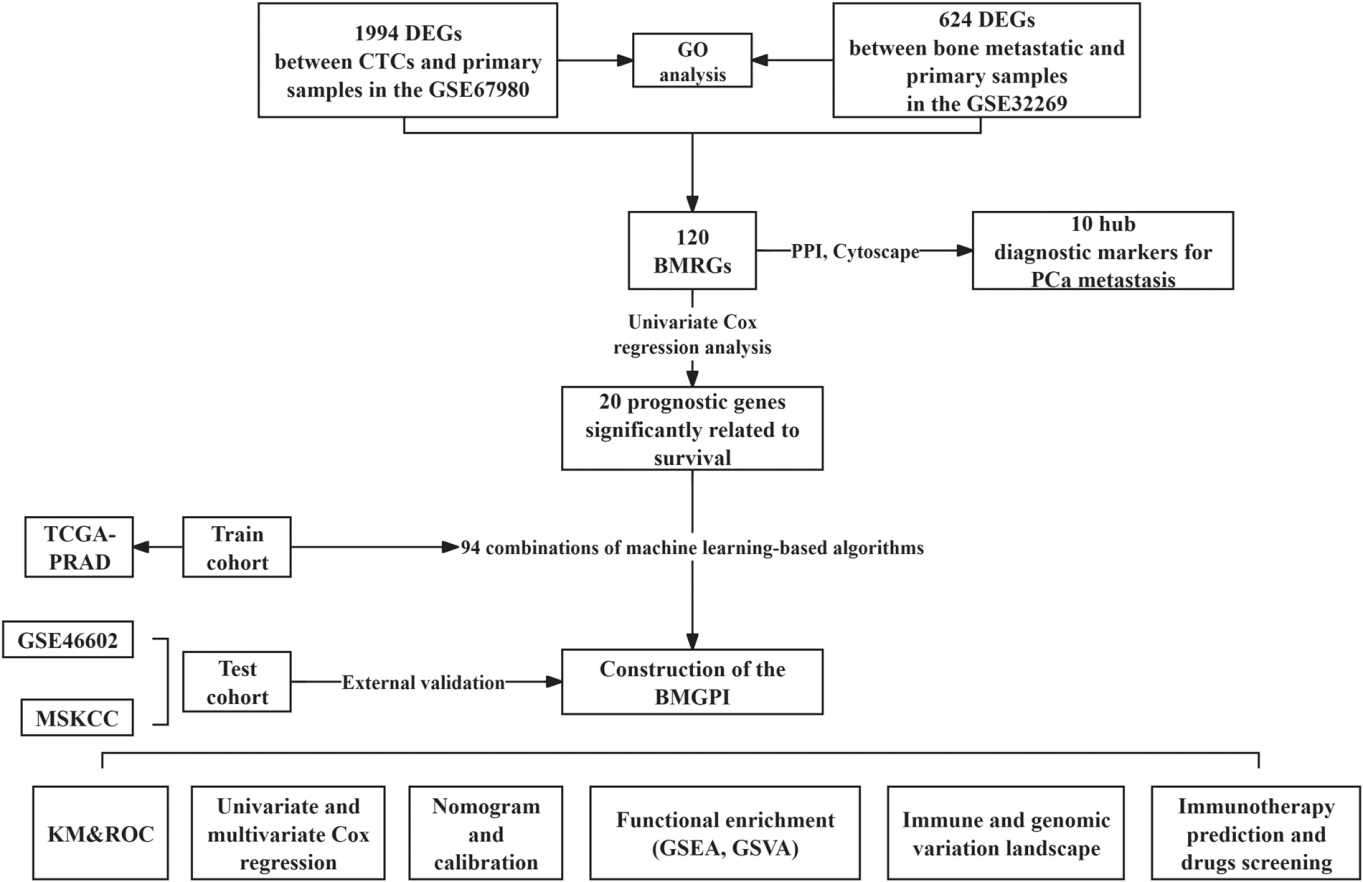
Flow chart of this research. DEGs, differentially expressed genes. CTCs, circulating tumor cells. BMRGs, bone metastasis-related genes. PPI, Protein–protein interaction. BMGPI, bone metastasis-related genes prognostic index. KM, Kaplan-Meier survival analysis. ROC, Receiver Operating Characteristic Curve. GSEA, gene set enrichment analysis. GSVA, gene set variation analysis.

Figure 1 illustrates the main flow of this study. First, we obtained 120 BMRGs by differential expression analysis as the basis for downstream analysis. Meanwhile, 10 diagnostic markers for PCa metastatic events were screened using PPI network and related plugins. Cox regression analysis was used to find prognostic genes to construct the model, which was then used to group patients. Functional enrichment, immune and mutational profiles, and immunotherapy efficacy were investigated in different groups to identify the ability of the model to discriminate between patients and to guide clinical practice.

**Fig. 2:**
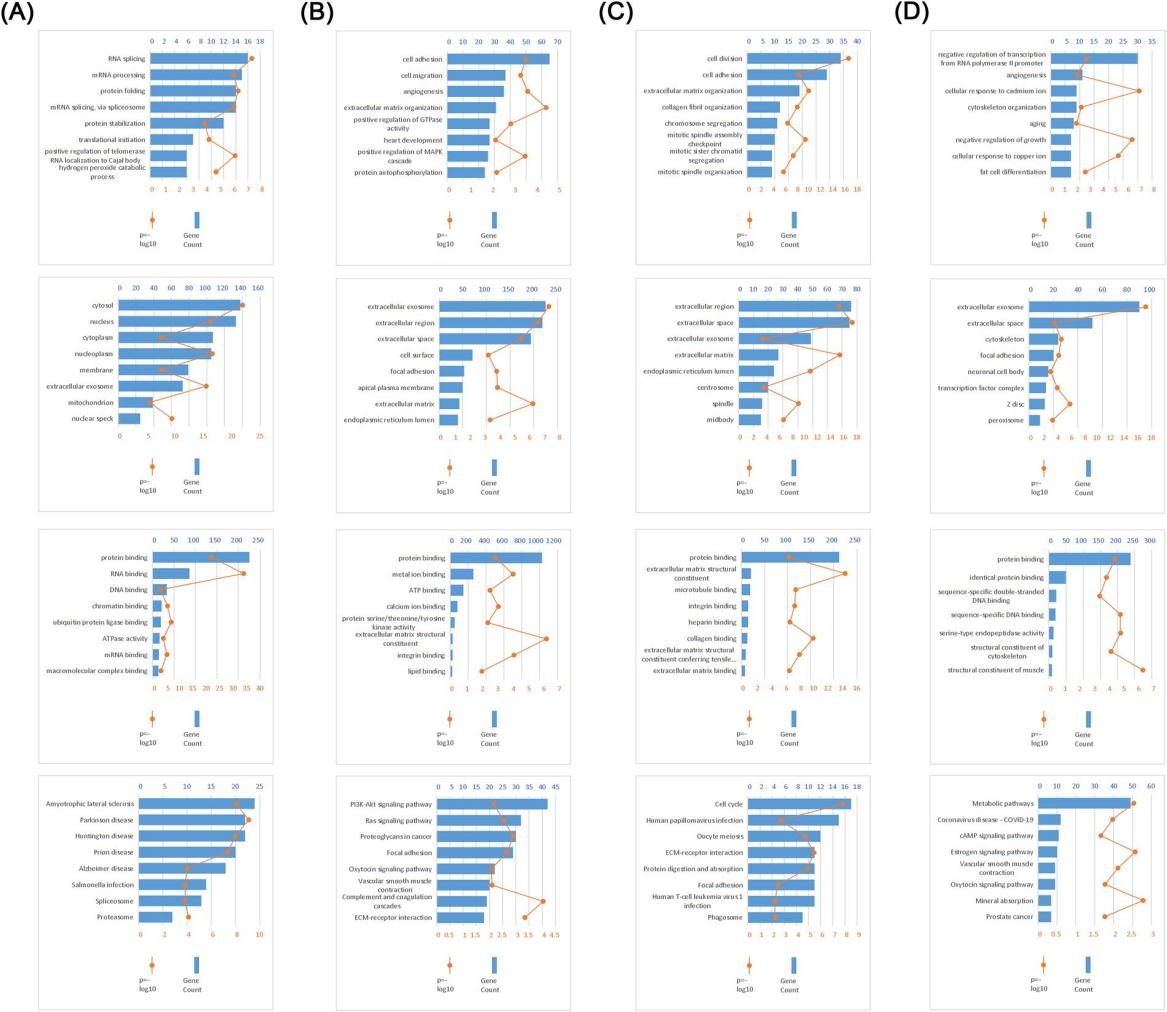
GO and KEGG analyses of 120 BMRGs. (**A**,**B**) GO (BP, CC, MF) and KEGG analysis of up-regulated and down-regulated DEGs in CTCs samples. (**C**,**D**) GO (BP, CC, MF) and KEGG analysis of up-regulated and down-regulated DEGs in bone metastasis samples.

A total of 1994 DEGs arose between CTCs and primary samples in the GSE67980 cohort (Table S1). In the GSE32269 cohort, we identified 624 DEGs between bone metastatic and primary samples (Table S2). Among the 1994 DEGs, 259 were up-regulated and 1,735 were down-regulated in CTCs. The up-regulated genes were mainly enriched in RNA splicing and protein folding and stabilization (Fig. 2A). Whereas down-regulated genes were associated with cell adhesion and angiogenesis, and were involved in PI3K-Akt signaling pathway and Ras signaling pathway (Fig. 2B). In PCa bone metastasis samples, there were 249 up-regulated and 345 down-regulated genes. The up-regulated genes were involved in cell division and cell cycle (Fig. 2C), and the down-regulated genes were enriched in angiogenesis, aging, and metabolic pathways (Fig. 2D). The intersection of DEGs from two cohorts was taken to obtain 120 core BMRGs related to bone metastasis.

### Identification of the hub module related to metastasis through the PPI network

**Fig. 3:**
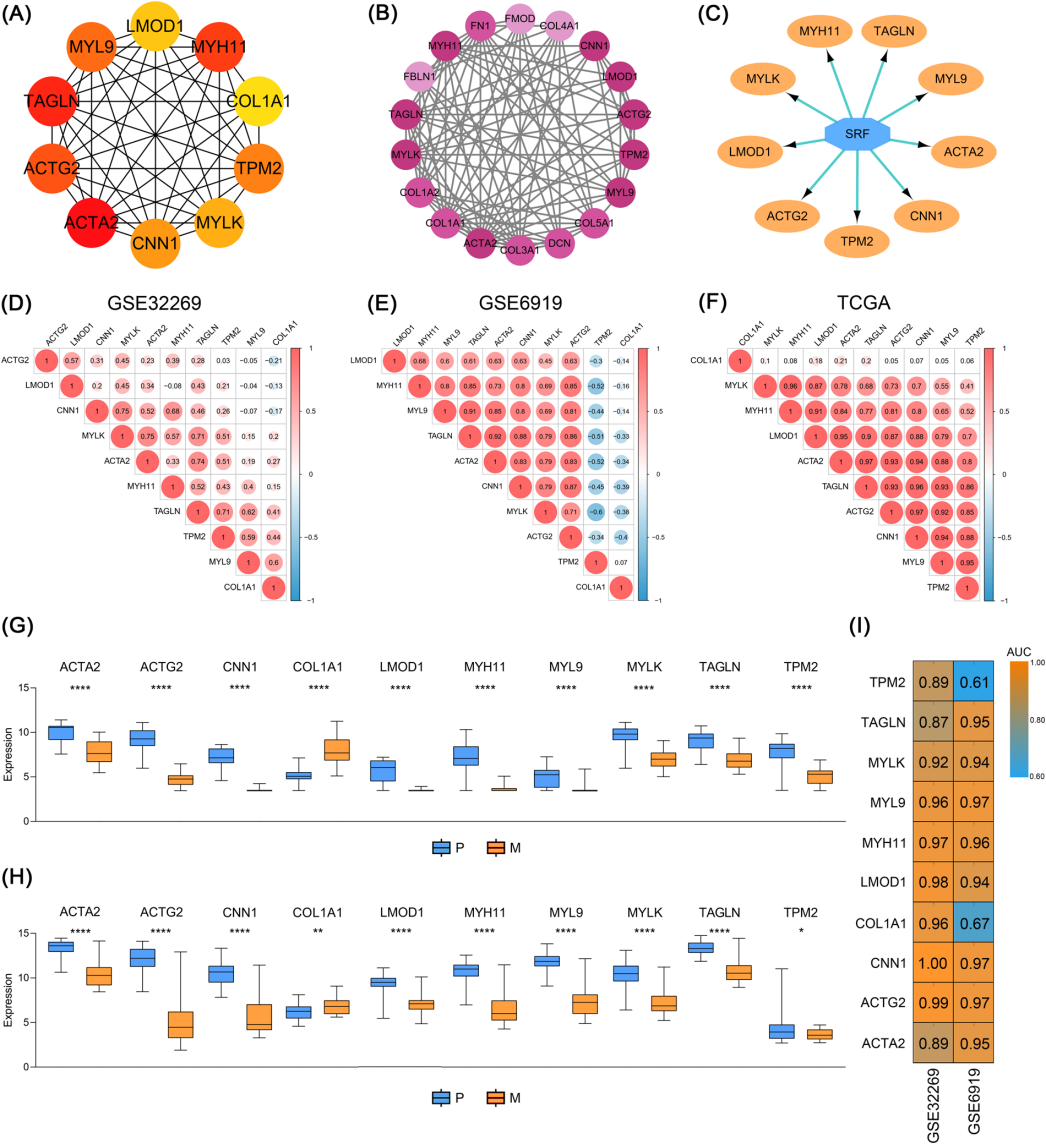
Screening hub genes by Cytoscape application. (**A**) Cytohubba and (**B**) MCODE plugins were utilized to screen for hub genes. (**C**) The gene regulation pattern was predicted by iRegulon plugin. Correlation analyses were performed for the ten hub genes in (**D**) GSE32269, (**E**) GSE6919, (**F**) and TCGA cohorts, respectively. Expression of these ten genes in (**G**) GSE32269 and (**H**) GSE6919 datasets. (**I**) AUC values demonstrated the predictive efficacy of hub genes for metastasis.

We constructed a PPI network based on 120 BMRGs and filtered the top ten genes with a degree greater than 10 using the MCC algorithm of the cytoHubba plugin, including *ACTA2*, *ACTG2*, *CNN1*, *COL1A1*, *LMOD1*, *MYH11*, *MYL9*, *MYLK*, *TAGLN* and *TPM2* (Fig. 3A). Additionally, to make the results more objective, we further screened three modules with MCODE plugin, and the most prominent module, which had a score of 11.294, 18 nodes, and 96 edges, is shown in Fig. 3B. This module still includes the ten genes mentioned above. Subsequently, the master regulators of ten intersecting genes were also explored by the iRegulon plugin and we found that nine of them were regulated by SRF (Fig. 3C).

The relevance of these ten hub genes was further analyzed. Except for *COL1A1*, the expression levels of the other nine genes exhibited a strong positive correlation (Fig. 3D–F), which was consistent with the predicted results demonstrated in Fig. 3C. To validate the diagnostic efficacy of these hub genes for tumor metastasis, we identified their expression patterns and calculated AUC values in both GSE32269 and GSE6919 datasets. These nine genes were less expressed in distant metastatic samples compared to primary samples, while *COL1A1* showed an opposite trend (Fig. 3G,H). As illustrated in Fig. 3I, the AUC values indicated superior diagnostic performance of the 10 hub genes.

### Development and validation of the BMGPI model based on integrative computational framework

**Fig. 4:**
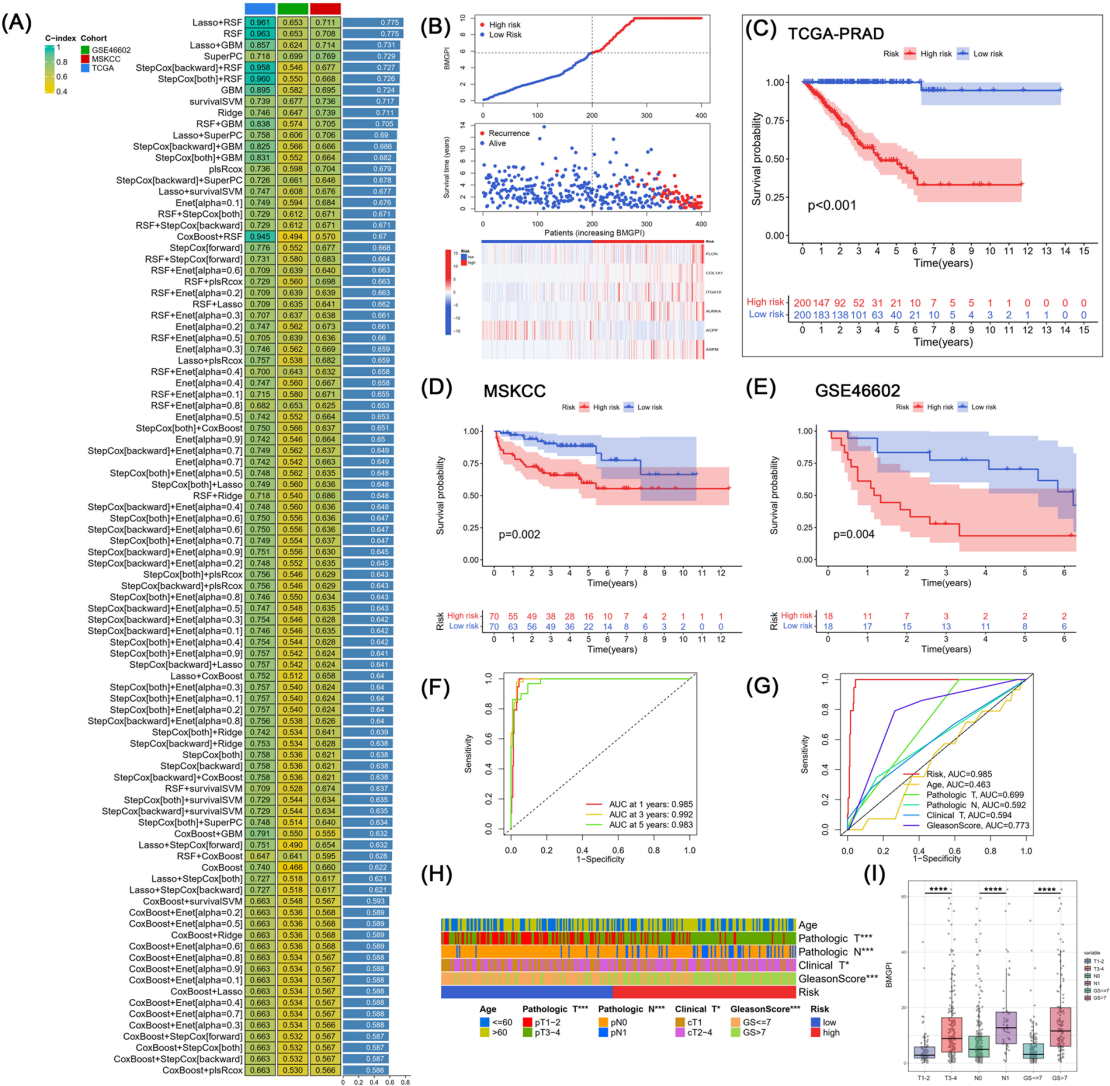
Construction and validation of BMGPI through the machine learning-based integrative program. (**A**) Ninety-four predictive models were applied to the training cohort and the validation cohort, and the C-index was calculated for each model across all cohorts. (**B**) The distribution of survival time, survival status, and the six genes comprising the model in the high- and low-risk groups. KM survival curves for risk groups in (**C**) TCGA, (**D**) MSKCC, (**E**) and GSE46602 cohorts. (**F**) Time-dependent and (**G**) clinical characteristic-related ROC curves. (**H**) Distribution of clinical characteristics in risk groups. (**I**) The difference in BMGPI between patients grouped by T stage, N stage, and Gleason score.

Firstly, univariate Cox analysis identified 20 prognostic genes from 120 BMRGs (*p* < 0.001). These 20 genes were subjected to 94 combinations of machine learning-based algorithms in order to construct a bone metastasis-related genes prognostic index (BMGPI). Afterwards, we further computed the C-index for the training and validation cohorts in each combined model (Fig. 4A). Interestingly, Lasso + RSF and RSF models were equally optimal among the 94 models, both of which led in terms of average C-index (0.775). Furthermore, both models also demonstrated good predictive capability in validation cohorts. Nevertheless, the Lasso + RSF model contained 11 genes, while the RSF model contained only 6 genes. Therefore, given the practicality and translational potential, we considered the RSF model as the optimal model as the basis for subsequent analysis.

Based on the random survival forest algorithm, each patient was assigned a risk score (BMGPI). Patients were stratified into high- and low-risk groups based on median scores. With increasing BMGPI, the survival of the patients became progressively worse (Fig. 4B). In addition, all five model genes except *ACPP* were highly expressed in the high-risk group. The survival curve in the training group suggested that the high-risk group was associated with a poor prognosis, which was validated in both the MSKCC and GSE46602 cohorts (Fig. 4C–E). The AUC values for 1-, 3-, and 5-year survival for the training group were 0.985, 0.992, and 0.983, respectively, as shown in Fig. 4F. Moreover, in Fig. 4G,risk had superior predictive efficacy compared to the Gleason score, T stage, and N-stage. Subsequently, we further assessed the distribution of various clinical characteristics in risk groups (Fig. 4H). The proportions of T-stage, N-stage and M-stage were significantly different between the two risk groups. BMGPI was also found to be higher in patients with advanced stages (Fig. 4I).

### BMGPI is an independent predictor for survival of PCa patients

**Fig. 5:**
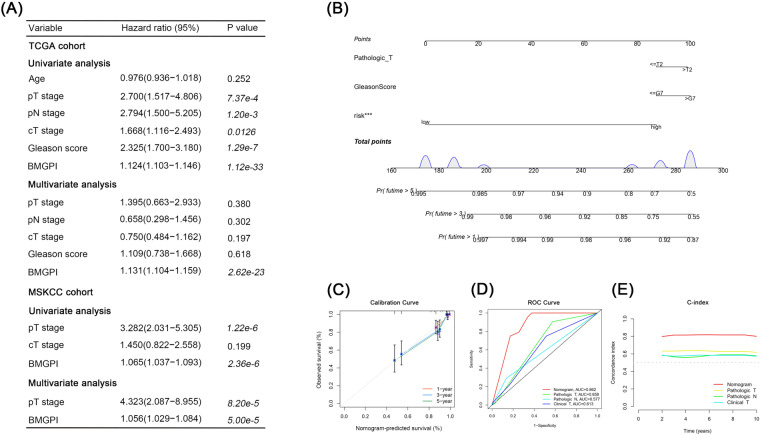
Construction of the nomogram relying on risk level and clinical parameters. (**A**) Univariate and multivariate Cox regression analysis for DFS in TCGA and MSKCC cohorts. (**B**) The nomogram based on T stage, Gleason score, and risk level. (**C**) Calibration plot for 1-, 3-, and 5-year survival. The comparison of (**D**) the AUC values and (**E**) C-index between the nomogram and other clinical characteristics.

Univariate and multivariate Cox analyses were utilized to evaluate the association of BMGPI and other clinical characteristics with prognosis of patients. BMGPI was identified as an independent prognostic factor for PRAD patients in both the TCGA and MSKCC cohorts (Fig. 5A). To extend the value of the model for clinical application, on the basis of risk level, pathologic T stage and Gleason score, we constructed a nomogram to predict 1-, 3- and 5-year survival (Fig. 5B). As demonstrated in Fig. 5C, the predicted survival of the nomogram was well consistent with the observed survival. The AUC and C-index also indicated that the constructed nomogram had more accurate and robust predictive ability compared with other variables (Fig. 5D,E).

### Functional enrichment in risk groups

**Fig. 6:**
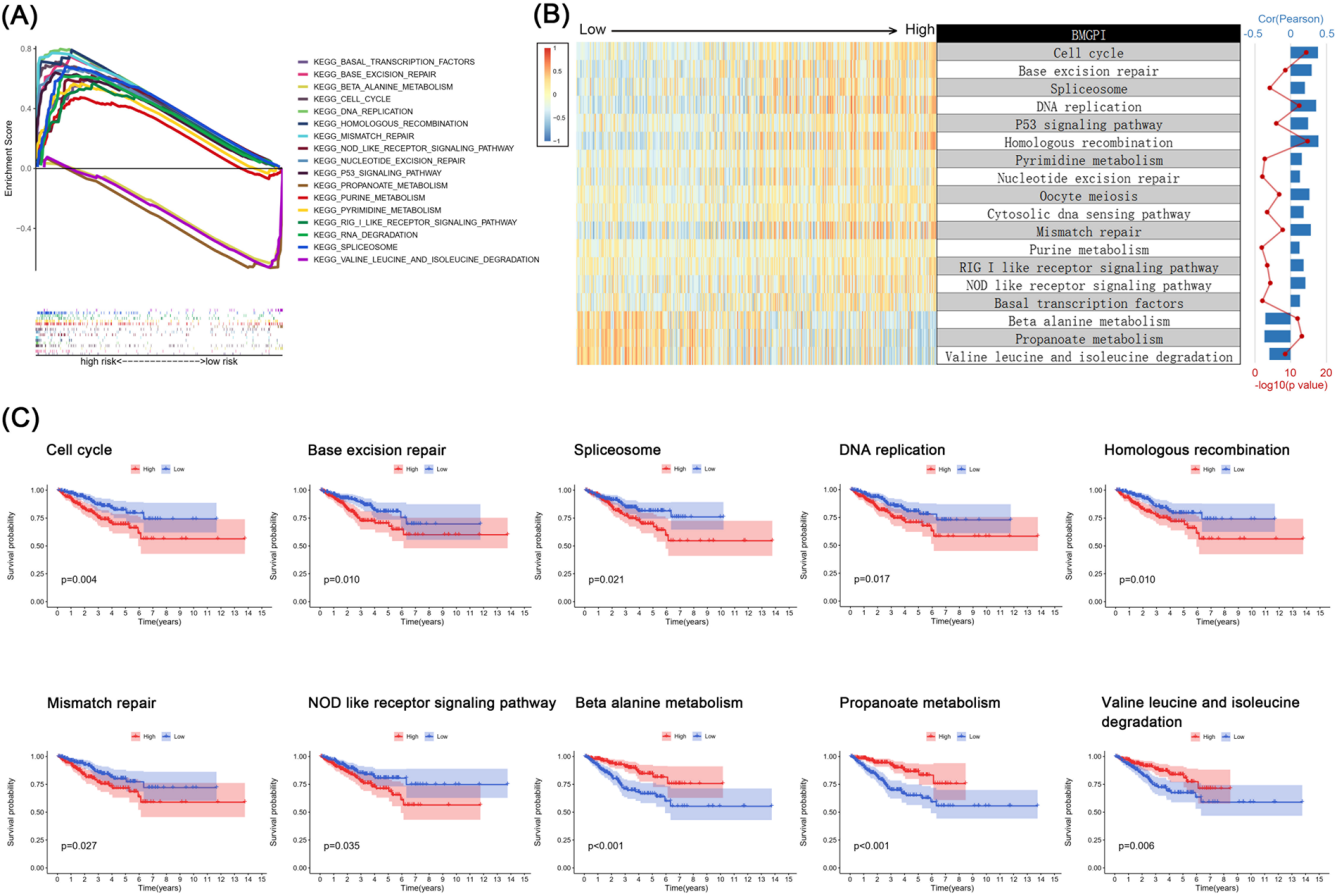
Function enrichment analysis. (**A**) The results of GSEA in risk groups. (**B**) Correlation analysis between BMGPI and immune enrichment scores. The colors of the heatmap represent the immune score calculated by GSVA for each patient. (**C**) Survival analysis revealed that GSVA scores for certain functional pathways were significantly associated with survival of patients.

According to GSEA, the pathways associated with the high-risk group mainly included cell cycle, base excision repair, DNA replication, P53 signaling pathway and homologous recombination, while several amino acid metabolic pathways were related to the low BMGPI (Fig. 6A). Then, we applied GSVA to further test the association of BMGPI with cellular pathways. The trend of the correlation between the BMGPI and enrichment scores remained consistent with the GSEA results (Fig. 6B). In order to determine whether these pathways influence the prognosis of patients, we performed a survival analysis based on the enrichment scores (Fig. 6C). Poor prognosis was correlated with pathways positively correlated with BMGPI, such as cell cycle, base excision repair, spliceosome, DNA replication, homologous recombination, mismatch repair and NOD like receptor signaling pathway. Whereas pathways positively correlated with BMGPI was correlated with good prognosis, such as beta alanine metabolism, propanoate metabolism and valine leucine and isoleucine degradation.

### Immune and genomic variation landscape in risk groups

**Fig. 7:**
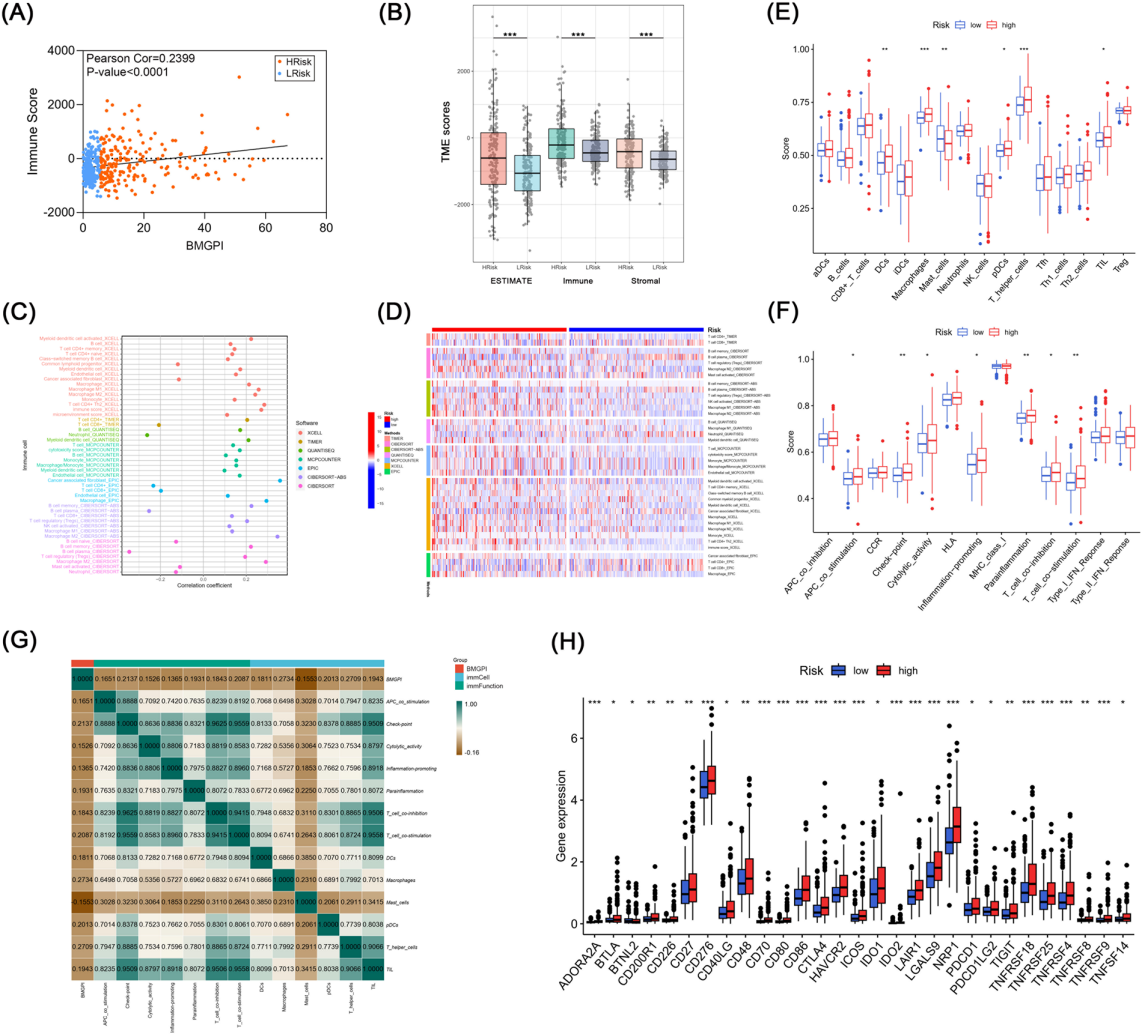
Immune landscape associated with BMGPI. (**A**) Correlation analysis between BMGPI and immune scores. (**B**) ESTIMATE score, immune score, and stromal score for risk groups. (**C**) The bubble plot showed the correlation coefficients between various types of immune cells and BMGPI. (**D**) The heatmap of immune cell abundance based on different software platforms. SsGSEA of (**E**) immune cells and (**F**) immune function. (**G**) The heatmap illustrated the correlation between the BMGPI and immune cell or function. (H) More immune checkpoints were activated in the high-risk group.

BMGPI was positively associated with immune scores (r = 0.2399, *p* < 0.0001) (Fig. 7A). Additionally, the high-risk group had higher immune activity compared to the low-risk group (Fig. 7B). The bubble plot demonstrated the correlation coefficients between BMGPI and different immune cell infiltration (Fig. 7C). The landscape of immune cells based on different methods in risk groups was presented in the heat map (Fig. 7D). The results of ssGSEA revealed that the TME of the high-risk group contained more dendritic cells (DCs), plasmacytoid dendritic cells (pDCs), macrophages, tumor infiltrating lymphocytes (TIL) and T helper cells, while the TME of the low-risk group had a higher content of mast cells (Fig. 7E). We also found that the high-risk group also showed more active in some immune functions, such as cytolytic activity and T cell co-stimulation (Fig. 7F). Correlation analysis of BMGPI with immune cells and immune function was visualized in Fig. 7G. Besides, a variety of immune checkpoints were enriched in the high-risk group (Fig. 7H).

**Fig. 8:**
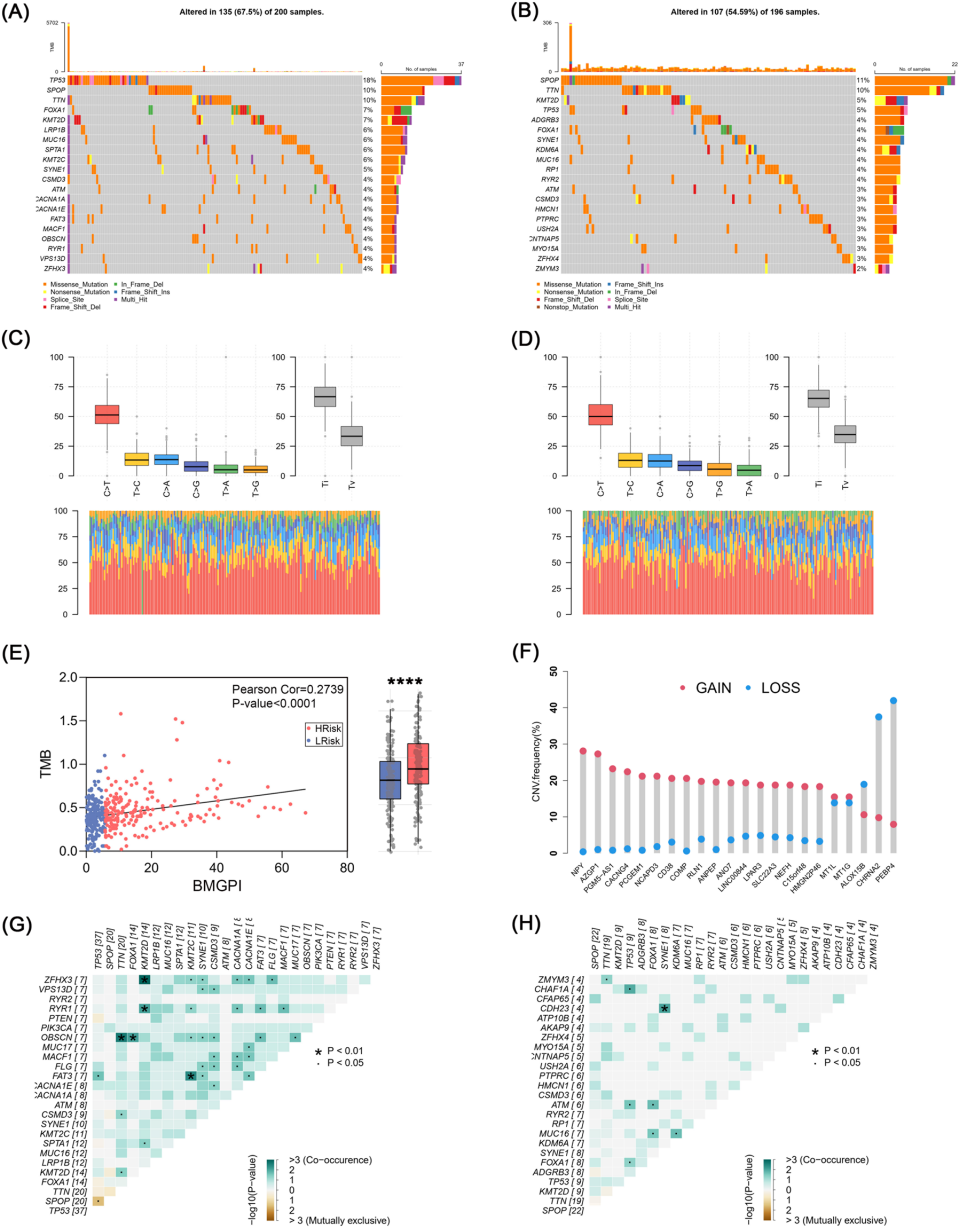
Genetic alterations in risk groups. Waterfall plots showing the clusters of genes with the highest frequency of somatic mutations in (**A**) high- and (**B**) low-risk groups. Summary of mutation patterns in (**C**) high- and (**D**) low-risk groups. (**E**) Scatter plot showing a positive correlation between the BMGPI and TMB. (**F**) CNV of DEGs between high- and low-risk groups. Heatmaps demonstrating the collinearity of mutations in the top 25 mutated genes of (**G**) high- and (**H**) low-risk groups.

We also depicted the mutation profiles of the high- and low-risk groups (Fig. 8A,B). *P53*, a well-known oncogene, was mutated significantly more frequently in the high-risk group (18%) than in the low-risk group (5%). Furthermore, as a key regulator of the androgen receptor (AR), mutations in *FOXA1* may cause alterations in the activity of transcription factors, promoting epithelial-mesenchymal transition (EMT) and cancer metastasis^26^. Its mutation frequency was 7% in the high-risk group, while it was only 4% in the low-risk group. The distribution of base mutation types was exhibited in Fig. 8C,D. BMGPI is positively correlated with tumor mutation burden (TMB) (r = 0.2739, *p* < 0.0001) (Fig. 8E). Moreover, we explored the copy number variation (CNV) of the differential genes between the two risk groups (|logFC| > 1, *p*. adj < 0.05). Apart from *ALOX15B*, *CHRNA2* and *PEBP4*, the main alteration in other genes was CNV gain (Fig. 8F). Finally, we analyzed the phenomenon of co-occurrence mutation in the high- and low-risk groups (Fig. 8G,H), which appeared more frequently in the high-risk group.

### Prediction of response to immunotherapy treatment

**Fig. 9:**
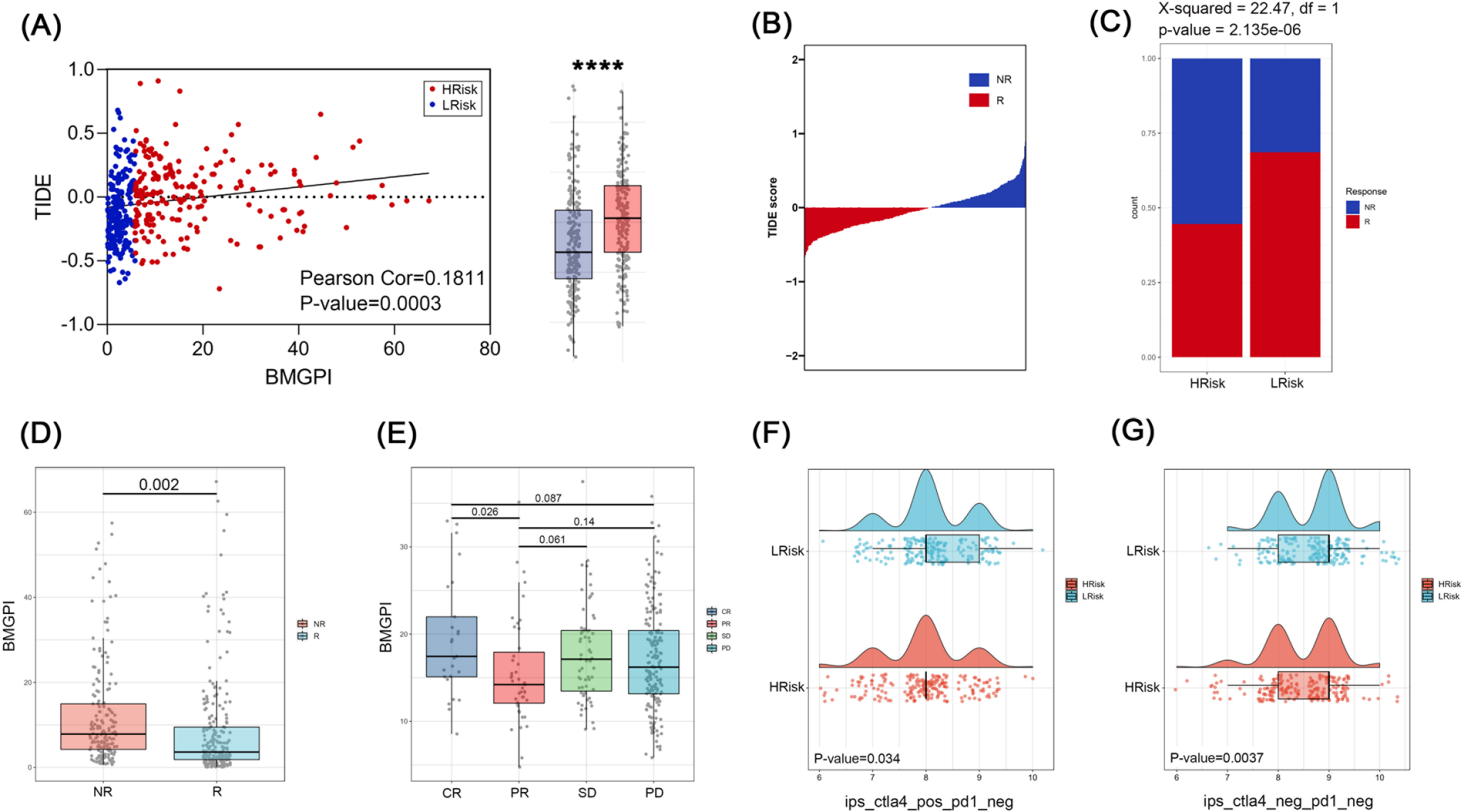
Immunotherapy sensitivity. (**A**) Scatter plot showing a positive correlation between the BMGPI and TIDE score. (**B**) The distribution of TIDE score in NR and R groups. (**C**) A graph plot depicting percentage of patients receiving immunotherapy who responded or did not respond in risk groups. (**D**) The difference in BMGPI between NR and R groups. (**E**) A box plot presenting the BMGPI of patients with CR, PR, SD, and PD in the IMvigor210 cohort. The IPS of the (**F**) CTLA4+/PD1- or (**G**) CTLA4-/PD1- treatment in risk groups. (**H**) Drug sensitivity analysis.

A TIDE score was calculated for each patient in the TCGA cohort to predict response to immunotherapy. TIDE scores increased with increasing BMGPI, indicating that the high-risk group may not respond well to immunotherapy (Fig. 9A). The chi-squared test suggested that the high-risk group responded to immunotherapy at a lower rate than the low-risk group (Fig. 9B,C). We further observed elevated BMGPI in the subgroup of patients who did not respond to immunotherapy (*p* = 0.002) (Fig. 9D). Interestingly, in the IMvigor210 cohort, we noted that BMGPI was higher in the CR group than in the PR group (*p* = 0.026), whereas there was no significant difference in BMGPI between the responding and non-responding groups (Fig. 9E). To further assess the efficacy of PD1 inhibitor and CTLA4 inhibitor in subgroups, we resorted to the IPS score from the TCIA database. In both CTLA4 (+) / PD1 (−) and CTLA4 (−) / PD1 (−) treatments, IPS scores were higher in the low-risk group, implying better efficacy (Fig. 9F,G). Finally, we screened 64 drugs with significantly different IC50s in the high and low risk groups, 10 of which were visualized in Fig. 9H, such as Bicalutamide and Paclitaxel.

## Discussion

### Research status and novelty of this study

In recent years, there have been numerous studies on PCa prediction models. You *et al*. constructed a new liquid-liquid phase separation-related index to predict biochemical recurrence and tumor microenvironment of PCa with the help of univariate Cox and Lasso analysis^27^. Similarly, Zhang *et al*. used Lasso-Cox method to develop a cancer-associated fibroblast-derived signature to predict PCa patients survival^28^. These models often rely on individual preferences in the selection of algorithms or lack validation across multiple datasets, resulting in overfitting or poor model performance. In this study, to obtain a stable and steady prognostic model, we developed a new computational framework containing 10 machine learning algorithms and their 94 combinations. The advantage of this framework is that it is based on various machine learning algorithms and their combinations to fit the model with consistent performance on PCa prognosis. Moreover, the combination of algorithms can reduce the dimensionality of the variables to simplify the model and improve its generalizability. ROC and C-index analyses showed that the BMGPI exhibited higher accuracy and stability compared to previous studies, indicating its great potential for clinical applications^27,28^.

### The main findings and relevant studies

In this study, we obtained 120 BMRGs by extracting common differential genes from the GSE67980 and GSE32269 datasets. GO functional enrichment analysis of these genes showed that genes up-regulated in CTCs or bone metastasis samples were mainly related to RNA splicing, cell cycle, while down-regulated genes were mainly enriched in cell adhesion, angiogenesis, and aging. Firstly, we screened the top ten highest ranked hub genes by cytoHubba and MCODE plugins, and master regulator analysis and correlation analysis indicated a high degree of consistency in expression and regulation patterns among the nine genes except collagen type I alpha 1 (*COL1A1*). They include proteins with contractile functions, such as alpha smooth actin 2 (*ACTA2*), gamma smooth actin 2 (*ACTG2*), calponin 1 (*CNN1*) and transgelin (*TAGLN*); myosin proteins, such as myosin light chain kinase (*MYLK*), myosin regulatory light polypeptide 9 (*MYL9*) and tropomyosin beta chain isoform 2 (*TPM2*)^29^. *ACTA2* and *ACTG2* are two cell type-specific isoforms of actin^30^. It has been revealed that *ACTA2* enables lung adenocarcinoma cells to acquire metastatic potential, and its down-regulation attenuates the invasive ability and mesenchymal characteristics of malignant cells^31^. A study found that *ACTG2* enhances the metastatic ability of hepatocellular carcinoma through the mediation of *NOTCH1*^32^. However, there have been few reports on the molecular mechanisms by which actin isoforms affect PCa metastasis. The anticancer effect of *MYH11* has been reported that can reverse the malignant phenotype of gastric cancer cells^33^. *COL1A1*, the only hub gene upregulated in metastatic samples, has been described to be involved in EMT and associated with proliferation, metastasis, and drug resistance in a variety of malignant tumors^34^. Furthermore, Zhu *et al*. observed that high expression of *COL1A1* played a driving role in PCa bone metastasis^35^. Although the association of some of these genes with PCa progression and metastasis is unclear, the AUC values demonstrate that they all have excellent predictive efficacy for PCa metastasis.

Subsequently, 20 prognostic genes were mined from 120 BMRGs by univariate Cox analysis. We constructed a novel combinatorial framework based on ten basic machine learning algorithms and applied it to the 20 genes to screen for an excellent bone metastasis-related prognostic signature. This framework worked well to reduce the size of the model thereby fitting a consensus risk model (BMGPI) containing only 6 genes (*FLCN*, *COL1A1*, *ITGA10*, *AURKA*, *ACPP* and *ASPM*). According to the median level of the BMGPI, the survival rate of the high-risk group was lower than that of the low-risk group in both the training and validation cohorts. Time-dependent and clinical characteristics-related ROC curves illustrated that the risk model had favorable predictive capability for patients’ prognosis at 1-, 3-, and 5-year, and its performance was superior to that of T stage, N stage and Gleason score. In addition, univariate and multivariate Cox analyses demonstrated that BMGPI was an independent prognostic factor for PCa patients in the TCGA-PRAD and MSKCC cohorts.

We also explored the biological processes involved in the high- and low-risk groups. Based on the GESA results, the high-risk group was mainly enriched in the cell cycle, p53 signaling pathway, NOD-like receptors (NLR) signaling pathway, and DNA replication and repair-related processes, while the low-risk group was associated with certain metabolic events. GSVA and correlation analyses further corroborated these findings. It has been found that enhancement of cell cycle activity inhibits anti-tumor immunity. Many pharmaceutical agents have been developed to promote anti-tumor immunity by inhibiting the cell cycle, such as kinase inhibitors of cyclin-dependent kinases 4 and 6 (CDK4/6)^36^. Interestingly, certain tumors are more sensitive to immune checkpoint blockade therapy when their cell cycle activity is inhibited, such as breast cancer and melanoma^37^. Additionally, p53, as a widely known tumor suppressor protein, can be activated and integrated a variety of stress processes, including oncogene activation, DNA damage, replication, and metabolic alterations^38^, which provides an explanation for the DNA replication and repair pathways likewise enriched in high-risk groups. According to recent evidence, NLR signaling pathway involves complicated crosstalk with cell death and autophagy^39^. Aberrant NLR signaling can trigger chronic inflammation which in turn drives genetic mutation and carcinogenesis. Besides, KM survival analysis showed that the above pathways were significantly associated with patient survival, indicating that altered activity of these pathways may affect survival outcomes in different subgroups.

Subsequently, we used multiple immune infiltration algorithms, including ESTIMATE, CIBERSORT, ssGSEA, EPIC, XCELL, TIMER, QUANTISEQ, and MCPCOUNTER, to depict the immune profile of risk groups. The high-risk group was observed to have higher immune score and more vibrant immunological landscape. The active immune cells in the high-risk group mainly consisted of CD4 + T cells, Tregs, M2 macrophages and pDC. CD4 + T cells can differentiate into Tregs and a variety of T helper (Th) cells. Tregs can suppress anti-tumor responses in the immune microenvironment through the production of interleukin-10 (IL-10) and transforming growth factor-β (TGF-β)^40^. The pro-tumorigenic activities of Th2 and Th17 have been reported in pancreatic and colon cancers^41–44^, and Th17 has also been mentioned to be associated with poor survival of patients with castration-resistant prostate cancer (CRPC)^45^. M2 macrophages, as a major subpopulation of tumor-associated macrophages (TAMs), play a key role in limiting immune responses, inducing angiogenesis and tissue repair^46^. It has been observed that pDC leads to a decrease in interferon α (IFN-α) and maintains the expansion of Tregs, promoting immune tolerance and tumor progression^47,48^. In contrast, CD8+T cells had a higher infiltrating abundance in the low-risk group. Through the specific recognition of antigens by major histocompatibility complex-I (MHC-I) on antigen-presenting cells (APCs), CD8+ T cells can be activated and subsequently produce cytotoxic effects leading to the death of tumor cells. Overall, the high-risk group had stronger pro-tumor and pro-inflammatory activities, while the low-risk group was more active in anti-tumor immunity.

Mutational events and TMB can contribute to tumor heterogeneity and affect immunotherapy response and prognosis of patients. It was observed in the mutation map that the mutation frequency of *FOXA1* was higher in the high-risk group than in the low-risk group. It has been shown that the mutation frequency of *FOXA1* in PCa is related to ethnicity, and this rate is higher in China than in the West^49^. *FOXA1* mutants can reshape chromatin structure in PCa. According to one study, its mutation reduces chromatin binding and promotes EMT, which increases cell growth in the absence of androgen^50^. Insertion deletions have been reported to be the most common type of *FOXA1* 3′ UTR mutation^51^, which is also reflected in our analyzed results. These mutations with PCa specificity are easily detected in liquid biopsies and are potential biomarkers for PCa^51^. Moreover, the survival outcomes of patients with different *FOXA1* mutation status need to be further investigated.

Most immunotherapies aim to reduce immune evasion by promoting immune activation, thus allowing T cells to eliminate cancer cells through toxic effects^52^. However, as mentioned earlier, CD8+ T cells, as the mainstay of anti-tumor immunity, were not enriched in the high-risk group, despite the fact that the high-risk group had a more active immune environment. Therefore, we further predicted the response of risk groups to immunotherapy. The TIDE algorithm revealed that the low-risk group was more sensitive to immunotherapy, which was in line with previous findings^53^. In addition, we explored the predictive effect of BMGPI in other tumor types. Unfortunately, due to tumor heterogeneity, BMGPI did not differ significantly between the CR/PR and SD/PD groups of patients with metastatic urothelial carcinoma taking anti-PD-1 drugs in the IMvigor210 cohort.

The study generated multiple gene markers and the prognostic model. Ten hub targets demonstrated excellent diagnostic performance for PCa metastasis in the GSE32269 and GSE6919 datasets based on the area under ROC. In addition, BMGPI correlated with T and N stage and Gleason score progression, which has important clinical implications. Almost all patients who die of PCa have symptoms of metastasis^2^. Early prediction of progression in PCa patients can improve clinical outcomes. This highlights the powerful clinical translational significance of metastatic diagnostic markers and BMGPI.

## Limitations and Future Validation

Admittedly, the study still has some flaws. First, the cohorts involved in this study were retrospective, and a prospective cohort will be needed to test the model in the future. Moreover, the roles of most bone metastasis-related biomarkers we screened in PCa remain unclear, and more *in vivo* and *in vitro* experiments are required to reveal their underlying biological mechanisms.

## Conclusions

In this study, based on bioinformatics and machine learning algorithms, we constructed a bone metastasis-related model for prognosis prediction in PCa patients. BMGPI is an independent predictor for survival of PCa patients. The high-risk group had high abundance of immune cells, high TMB, and poor response to immunotherapy. This BMGPI model is a promising tool to optimise decision-making for individual PCa patients.

### Supplementary information

Supplementary Table S1-5

## Data Availability

The analysis results associated with this paper is available on figshare (10.6084/m9.figshare.24935922.v1)^54^.

## Code availability

The R code used in the analysis of the data is available on figshare (10.6084/m9.figshare.24935922.v1)^54^.

## References

[1] Yamada Y, Beltran H (2021). The treatment landscape of metastatic prostate cancer. Cancer Lett..

[2] Long M (2021). Alendronate-functionalized hypoxia-responsive polymeric micelles for targeted therapy of bone metastatic prostate cancer. J Control Release..

[3] de Bono JS (2010). Prednisone plus cabazitaxel or mitoxantrone for metastatic castration-resistant prostate cancer progressing after docetaxel treatment: a randomised open-label trial. Lancet..

[4] Hofman MS (2018). [(177)Lu]-PSMA-617 radionuclide treatment in patients with metastatic castration-resistant prostate cancer (LuPSMA trial): a single-centre, single-arm, phase 2 study. Lancet Oncol..

[5] Ren C (2023). Metabolic syndrome-related prognostic index: Predicting biochemical recurrence and differentiating between cold and hot tumors in prostate cancer. Front Endocrinol (Lausanne)..

[6] Sethi N, Kang Y (2011). Unravelling the complexity of metastasis - molecular understanding and targeted therapies. Nat Rev Cancer..

[7] Caramel J (2013). A switch in the expression of embryonic EMT-inducers drives the development of malignant melanoma. Cancer Cell..

[8] Kim YN, Koo KH, Sung JY, Yun UJ, Kim H (2012). Anoikis resistance: an essential prerequisite for tumor metastasis. Int J Cell Biol..

[9] Yu M (2012). RNA sequencing of pancreatic circulating tumour cells implicates WNT signalling in metastasis. Nature..

[10] Tulpule V, Morrison GJ, Falcone M, Quinn DI, Goldkorn A (2022). Integration of Liquid Biopsies in Clinical Management of Metastatic Prostate Cancer. Curr Oncol Rep..

[11] Miyamoto DT (2015). RNA-Seq of single prostate CTCs implicates noncanonical Wnt signaling in antiandrogen resistance. Science..

[12] Cai C (2013). ERG induces androgen receptor-mediated regulation of SOX9 in prostate cancer. J Clin Invest..

[13] Tomczak K, Czerwinska P, Wiznerowicz M (2015). The Cancer Genome Atlas (TCGA): an immeasurable source of knowledge. Contemporary oncology (Poznan, Poland)..

[14] Zehir A (2017). Mutational landscape of metastatic cancer revealed from prospective clinical sequencing of 10,000 patients. Nat Med..

[15] Huang DW (2007). The DAVID Gene Functional Classification Tool: a novel biological module-centric algorithm to functionally analyze large gene lists. Genome Biol..

[16] Subramanian A (2005). Gene set enrichment analysis: A knowledge-based approach for interpreting genome-wide expression profiles. Proc Natl Acad Sci USA.

[17] Hänzelmann S, Castelo R, Guinney J (2013). GSVA: gene set variation analysis for microarray and RNA-Seq data. BMC Bioinformatics..

[18] Li TW (2020). TIMER2.0 for analysis of tumor-infiltrating immune cells. Nucleic Acids Res..

[19] Szklarczyk D (2023). The STRING database in 2023: protein-protein association networks and functional enrichment analyses for any sequenced genome of interest. Nucleic Acids Res..

[20] Shannon P (2003). Cytoscape: a software environment for integrated models of biomolecular interaction networks. Genome Res..

[21] Yu YP (2004). Gene expression alterations in prostate cancer predicting tumor aggression and preceding development of malignancy. J Clin Oncol..

[22] Mortensen MM (2015). Expression profiling of prostate cancer tissue delineates genes associated with recurrence after prostatectomy. Sci Rep..

[23] Jiang P (2018). Signatures of T cell dysfunction and exclusion predict cancer immunotherapy response. Nat Med..

[24] Charoentong P (2017). Pan-cancer Immunogenomic Analyses Reveal Genotype-Immunophenotype Relationships and Predictors of Response to Checkpoint Blockade. Cell Rep..

[25] Yang W (2013). Genomics of Drug Sensitivity in Cancer (GDSC): a resource for therapeutic biomarker discovery in cancer cells. Nucleic Acids Res..

[26] Teng M, Zhou S, Cai C, Lupien M, He HH (2021). Pioneer of prostate cancer: past, present and the future of FOXA1. Protein Cell..

[27] You Q (2023). A Liquid-Liquid Phase Separation-Related Index Associate with Biochemical Recurrence and Tumor Immune Environment of Prostate Cancer Patients. Int J Mol Sci..

[28] Zhang R, Liu F (2022). Cancer-associated fibroblast-derived gene signatures predict radiotherapeutic survival in prostate cancer patients. J Transl Med..

[29] Mele V (2022). Identification of TPM2 and CNN1 as Novel Prognostic Markers in Functionally Characterized Human Colon Cancer-Associated Stromal Cells. Cancers (Basel)..

[30] Suresh R, Diaz RJ (2021). The remodelling of actin composition as a hallmark of cancer. Transl Oncol..

[31] Lee HW (2013). Alpha-smooth muscle actin (ACTA2) is required for metastatic potential of human lung adenocarcinoma. Clin Cancer Res..

[32] Wu Y (2017). Identification of ACTG2 functions as a promoter gene in hepatocellular carcinoma cells migration and tumor metastasis. Biochem Biophys Res Commun..

[33] Wang J (2021). Interaction between DNMT3B and MYH11 via hypermethylation regulates gastric cancer progression. BMC Cancer..

[34] Li X (2022). COL1A1: A novel oncogenic gene and therapeutic target in malignancies. Pathol Res Pract..

[35] Zhu Z (2020). Identifying the key genes and microRNAs in prostate cancer bone metastasis by bioinformatics analysis. FEBS Open Bio..

[36] Li J, Stanger BZ (2020). Cell Cycle Regulation Meets Tumor Immunosuppression. Trends Immunol..

[37] Goel S (2017). CDK4/6 inhibition triggers anti-tumour immunity. Nature..

[38] Hernández Borrero LJ, El-Deiry WS (2021). Tumor suppressor p53: Biology, signaling pathways, and therapeutic targeting. Biochim Biophys Acta Rev Cancer..

[39] Saxena M, Yeretssian G (2014). NOD-Like Receptors: Master Regulators of Inflammation and Cancer. Front Immunol..

[40] Shitara K, Nishikawa H (2018). Regulatory T cells: a potential target in cancer immunotherapy. Ann N Y Acad Sci..

[41] Chen J (2018). E2F1/SP3/STAT6 axis is required for IL-4-induced epithelial-mesenchymal transition of colorectal cancer cells. Int J Oncol..

[42] He S (2011). Distribution and clinical significance of Th17 cells in the tumor microenvironment and peripheral blood of pancreatic cancer patients. Int J Mol Sci..

[43] Prokopchuk O, Liu Y, Henne-Bruns D, Kornmann M (2005). Interleukin-4 enhances proliferation of human pancreatic cancer cells: evidence for autocrine and paracrine actions. Br J Cancer..

[44] Tosolini M (2011). Clinical impact of different classes of infiltrating T cytotoxic and helper cells (Th1, th2, treg, th17) in patients with colorectal cancer. Cancer Res..

[45] Derhovanessian E (2009). Pretreatment frequency of circulating IL-17+ CD4+ T-cells, but not Tregs, correlates with clinical response to whole-cell vaccination in prostate cancer patients. Int J Cancer..

[46] Duan Z, Luo Y (2021). Targeting macrophages in cancer immunotherapy. Signal Transduct Target Ther..

[47] Diamond MS (2011). Type I interferon is selectively required by dendritic cells for immune rejection of tumors. J Exp Med..

[48] Palucka K, Banchereau J (2012). Cancer immunotherapy via dendritic cells. Nat Rev Cancer..

[49] Li J (2020). A genomic and epigenomic atlas of prostate cancer in Asian populations. Nature..

[50] Adams EJ (2019). FOXA1 mutations alter pioneering activity, differentiation and prostate cancer phenotypes. Nature..

[51] Annala M (2018). Frequent mutation of the FOXA1 untranslated region in prostate cancer. Commun Biol..

[52] Schumacher TN, Schreiber RD (2015). Neoantigens in cancer immunotherapy. Science..

[53] Xue L (2021). Using Immune-Related lncRNA Signature for Prognosis and Response to Immunotherapy in Cutaneous Melanoma. Int J Gen Med..

[54] Ren C (2024). figshare.

